# Introducing stochastic data envelopment analysis models for evaluating circular economy performance in European countries: pathways towards sustainability

**DOI:** 10.1007/s00291-025-00841-y

**Published:** 2026-03-31

**Authors:** Behrouz Arabi, Mehdi Toloo, Hajar Farnoudkia, Bing Xu

**Affiliations:** 1 School for Business and Society, University of York, York, UK; 2 Centre for Financing a Sustainable Future, Heriot-Watt University, Edinburgh, UK; 3 Management Discipline, Surrey Business School, University of Surrey, Guildford, UK; 4 Department of Systems Engineering, Faculty of Economics, Technical University of Ostrava, Ostrava, Czechia; 5 Department of Business Administration, Başkent University, Ankara, Turkey

**Keywords:** Network data envelopment analysis, Chance constrained programming, Circular economy, European countries

## Abstract

This paper addresses a critical gap in circular economy (CE) research by introducing a novel methodological framework that integrates Network Data Envelopment Analysis (NDEA) with a chance-constrained programming. The proposed approach captures the interrelated dynamics of economic production and waste treatment subsystems, while accounting for stochastic variables and data uncertainties to provide robust CE efficiency estimates. Using data from 26 European (EU) countries from 2013 to 2020, our results reveal that achieving CE efficiency requires a balanced focus on economic production and waste management. Although strong economic output can support circularity, waste treatment efficiency often plays a decisive role in determining overall CE performance. Moreover, we find that economic size does not necessarily translate into circular efficiency, whilst large economies may face challenges with effective waste management and resource recovery despite their economic status. The proposed approach offers policymakers and practitioners a robust empirical framework to guide CE improvements, particularly in regions where environmental practices lag behind economic achievements. Stronger incentives and regulatory measures are recommended to enhance circular activities within the EU and foster greater circular efficiency across countries.

## Introduction

The world faces an increasingly complex and interconnected set of challenges spanning socio-economic and environmental dimensions. The rapid acceleration of climate change, driven by escalating greenhouse gases (GHG) emissions, presents an urgent threat to the environment (NOAA 2023). At the same time, global economic downturns and the depletion of resources exacerbate this intricate landscape. In Europe, these challenges are particularly pronounced, with household expenditures surged by 69% from €4.3 trillion to €7.3 trillion (EEA 2023a), yet only 11.7% of the 6.95 billion tonnes of material used within the EU economy are recovered and reintegrated into the economic cycle. Moreover, nearly one-quarter of the resources employed within the EU are imported from external sources (EEA 2020).

A promising solution to these challenges lies in the adoption of the CE, a paradigm aimed at decoupling economic growth from resource consumption while fostering value creation through innovative consumption and production patterns (James et al. 2023). The European Commission has been a strong proponent of this approach through initiatives like the *Roadmap to a Resource Efficient Europe* (2011) and the *EU Action Plan for the CE* (2015), which are designed to enhance resource efficiency and facilitate the transition towards circularity (Domenech and Bahn-Walkowiak 2019). However, despite growing interest among policymakers, researchers and practitioners, transitioning from a linear to a circular economy remains challenging for societies that are deeply rooted in traditional production practices.

Achieving sustainability in CE initiative requires thoughtful design, with sustainability as a core principle, coupled with ongoing rigorous evaluation and monitoring of sustainability performance (Kravchenko et al. 2020). Standardised measurement tools are essential for evidence-based management (Davies 2002), and there is broad consensus among academics, industry practitioners, and policymakers on the need for CE-specific tools to track progress at national, regional, and sectoral levels (Saidani et al. 2019). A thorough review of the literature (e.g., Sassanelli et al. 2019) reveals several prevalent methodologies for evaluating CE performance, such as Life Cycle Assessment (LCA), Material Cost Analysis (MCA), Material Flow Cost Accounting (MFCA), Multi-Criteria Decision Making (MCDM), and Data Envelopment Analysis (DEA). Among these, DEA has gained particular popularity for assessing circular systems’ performance due to its ability to treat decision-making units (DMUs) as black boxes, focusing on their inputs and outputs while bypassing the internal complexities of processes involved (Gennitsaris et al. 2023; Fan and Fang 2020; Wu et al. 2014; Lombardi et al. 2021). Recent advancements have led to the NDEA models (Hatami-Marbini et al. 2022; Azadi et al. 2024), which account for multi-stage DMU processes (Färe et al. 2007). The NDEA model with feedback has been applied in several studies to assess CE performances (Xu et al. 2021; Torabi Golsefid and Salahi 2023; Nematizadeh et al. 2019; Ding et al. 2020; Sun et al. 2019).

Despite these advancements, two key challenges persist in CE evaluation. First, the lack of a standardised CE definition, combined with the diversity of associated practices create inconsistencies in measurement methods. This is evidenced by over 114 distinct CE-related definitions (Kirchherr et al. 2017) and more than 270 distinct measurement indicators reported in previous studies (Kravchenko et al. 2020). The second challenge is rooted in the limitations of data utilisation. CE systems are complex, encompassing multifaceted supply chains that span collection, sorting, remanufacturing, and distribution stages. Each stage can potentially introduce errors in data collection, creating vulnerabilities that current assessment tools often fail to address. Without accounting for these uncertainties, evaluation results risk being incomplete or misleading. Although the broader CE assessment literature has explored the incorporation of uncertain variables, particularly within DEA and NDEA methodologies, the treatment of uncertainty in relation to CE drivers remains underdeveloped (Vanson et al. 2022; Izadikhah et al. 2019; Izadikhah 2020; Peykani et al. 2022).

Recognising the pivotal role of CE practices in shaping both the EU's internal dynamics and its global influence, this paper addresses the issue of uncertainty in CE measures for EU countries using data from 2013 to 2020. First, we establish a robust framework conceptualising the CE system as two interlinked subsystems: *Economic Production Subsystem (EPS)* and *Waste Treatment Subsystem (WTS)*. These subsystems form the backbone of material flow within the CE model, embedding sustainability principles into economic and environmental processes. Second, we introduce the innovative concept of circular disposability, establishing a robust and transformative foundation for advancing CE efficiency measurement models. Third, we prove that in a circular system, as modelled, the efficiency of the EPS is contingent on the efficiency of the WTS; however, the converse does not hold. This asymmetry underscores the intrinsic interdependence between the EPS and WTS. Finally, we examine the inherent data uncertainties presented in CE metrics for EU countries. To address these challenges, we propose an innovative chance-constrained NDEA model. This model explicitly captures the interconnectedness between EPS and WTS while addressing data variability.

The remainder of this paper is organised as follows. Section 2 provides a brief literature review on CE assessment. Section 3 presents the research framework, developed using the Design Science Research Methodology (DSRM). Section 4 outlines the CE measurement framework and analyses the data used in the analysis. Section 5 introduces the proposed NDEA model, detailing its features. Section 6 discusses our key findings, highlighting insights from the analysis. Finally, Sect. 7 concludes with a comprehensive summary of the findings and the implications of our research.

## Literature review

This section reviews existing literature on measuring CE performance, with a particular focus on DEA and NDEA methodologies, as well as the CE performance in EU countries.

### CE performance measurement

The concept of the CE has become central to industrial and environmental policies in many countries, such as China (Liu et al. 2009), Japan (MOE 2010) and the UK (Hill 2016). After the 2008 financial crisis, there has been growing interest in ‘*green growth*’, underscoring resource productivity and efficiency under the motto of achieving *“more with less”*. In response, the European Commission introduced its vision for a ‘*Resource Efficient Europe*’, which became a key pillar of the ‘*Europe 2020 Strategy*’ (Huhtala 2015). CE aims to promote sustainable economic growth through material recycling, resource security, pollution reduction, and adherence to the 9R principles: refuse, rethink, reduce, reuse, repair, refurbish, remanufacture, repurpose, recycle and recover (Hunger et al. 2024). Despite progress, critics highlight inefficiencies in EU waste policy, with concerns about its limited effectiveness in driving waste reduction (Giannakitsidou et al. 2020; Potting et al. 2544). In addition, current trajectories indicate that the EU might fall short of its ambitious target to double the circular material use rate by 2030 (EEA 2023b).

A significant body of research has sought to address ambiguities in CE performance indicators. Notably, Saidani et al. (2019) developed a taxonomy comprising 55 indicators, categorised into ten dimensions based on CE implementation levels, CE loops as in Ellen MacArther Foundation’s butterfly diagram, and development context. In a parallel vein, Kravchenko et al. (2020) created an extensive database of over 270 leading indicators, systematically classified based on sustainability dimensions, business processes, and CE strategies. For firm-level CE measurement, Vinante et al. (2021) and Negri et al. (2021) provide critical insights, while Sassanelli et al. (2019) provides an in-depth review of the state-of-the-art methodologies.

### Employing DEA for CE performance measurement

DEA has emerged as a prominent method for performance measurement (Xiong et al. 2011; Zeng et al. 2009), with its applications spanning various domains, including CE performance. Since its inception, the DEA framework has undergone significant advancements, such as the introduction of non-discretionary environmental adjustments to account for external factors beyond the control of decision-makers (Charnes et al. 1978; Charnes, et al. 1986). The use of DEA in CE performance assessment gained its popularity with a study evaluating a city’s CE progress (Li and Xu 2008). This study paved the way for numerous research initiatives exploring CE across various contexts, such as analysing the impact of CE on carbon intensity, addressing wind turbine end-of-life issues, conducting provincial CE assessments, assessing recycling and material use in municipal waste management, and examining CE practices in coal-fired power plants (Gennitsaris et al. 2023; Wu et al. 2014; Xiong et al. 2011; Zeng et al. 2009; Chen 2022; Marques and Teixeira 2022). These applications highlight DEA’s flexibility and effectiveness in addressing the multifaceted challenges of CE. Recent studies have further expanded DEA's applicability by integrating it with other models (Sassanelli et al. 2019). For instance, DEA combined with Tobit regression model was used to assess recycling and renewable energy across EU countries (Horvat et al. 2023). DEA and Malmquist productivity index were applied to analyse resource use efficiency in the CE policies of OECD countries (Škrinjarić 2020).

Note that transitioning from traditional linear supply chains to circular systems involves integrating waste treatment systems with feedback loops, adding significant complexity. To address these challenges, NDEA models have emerged as particularly effective tools for evaluating CE performances (Färe et al. 2007; Boloori et al. 2016; Tone and Tsutsui 2009). NDEA models, especially those incorporating feedback mechanisms, are critical in CE performance measurement. The intricate interplay between inputs and outputs in the CE systems requires approaches that enable simultaneous improvements of both. This is where Directional Distance Function (DDF) NDEA models excel. First introduced in Charnes et al. (1986), the DDF approach allows for the concurrent optimisation of inputs and outputs, thereby enhancing the overall effectiveness of NDEA methodologies. Further exploration of DDF-NDEA models in Färe et al. (2007) provided a comprehensive understanding of their intricacies and reinforced their importance in addressing the inherent complexity of CE systems.

NDEA frameworks have continued to evolve, with refinements adding new dimensions and enhancing their analytical ability (Färe and Grosskopf 1996). A notable development was the creation of a taxonomy that categorises NDEA models into classes, encompassing standard game-theoretic centralised global optimal solution, and feedback models (Cook and Zhu 2014). This taxonomy provided a structured framework to deepen understanding of the multifaceted nature of NDEA. Subsequent studies further extended this classification by introducing additional dimensions, improving the versatility and scope of NDEA methodologies (Kao 2009, 2014, 2016). For example, a game meta-frontier NDEA approach has been introduced to evaluate technology heterogeneity and efficiency in China’s CE, utilising a leader–follower dynamic to assess production and environmental performance (Sun et al. 2019). An extended Malmquist index based on cooperative game NDEA model was applied to study the dynamic evolution of industrial CE, including marine CE systems in China (Ding et al. 2020).

### CE performance measurement across different levels

One strand of studies examines CE performance on across micro-, meso- and macro-levels. At the *micro-level*, the focus is on individual products and companies. For instance, Mesa et al. (2020) evaluated material durability and environmental footprints during the product development phase. Similarly, James et al. (2023) used a directed graph methodology to monitor CE performance in service-oriented businesses.

At the *meso-level*, the emphasis shifts to sectors and industrial parks. For example, Han et al. (2017) conducted an in-depth evaluation of CE implementation within a Chinese aluminium industrial park, highlighting structural upgrades, functional enhancements, and facility development. Similarly, Wang et al. (2021) used DEA models to analyse the efficiency of industrial parks in China focusing on energy savings and pollution reduction. Further studies have explored sector-specific CE performance, such as in food (Pagotto and Halog 2016), constructions (Akanbi et al. 2018), plastics (Huysman et al. 2017) (Robaina et al. 2020), and textiles (Angelis-Dimakis et al. 2016).

At the *macro-level*, the focus broadens to regional or national evaluations. Due to the comprehensive historical CE performance data provided by the Eurostat database, it attracts many studies to assess CE performance in EU countries. For example, one of the first studies, Busu and Busu (2018) applied a Shannon Entropy-based evaluation algorithm to model CE processes in the EU, creating a composite indicator through a weighted summation of individual indicators. Weights reflected the importance of each indicator in shaping the composite measure. Another study, Škrinjarić (2020) evaluated CE framework using essential variables outlined by the European Commission, with rigorous comparisons ensuring the robustness of findings.

In terms of the methodologies, various methodologies have been employed to assess CE efficiency, with DEA emerging as a widely used approach, as summarised in Table 1. For instance, Giannakitsidou et al. (2020) was the first to apply DEA to measure the performance EU countries in transitioning to a CE, evaluating both environmental and CE performance. Similarly, Potkány et al. (2024) ranked EU countries’ recycling and material use efficiency using DEA models, noting that relying solely on metrics like recycling, or cyclical material use rate for municipal solid waste can lead to overestimations or underestimations of a country’s true performance. For example, low waste generation, an often-overlooked factor, may indicate a country’s economic and social progress.

**Table 1:** CE performance measurement inputs and outputs

Title of the reference	Year	Model	Inputs	Outputs	Countries
Modelling the circular economy processes at the EU level using an evaluation algorithm based on Shannon entropy (Busu and Busu 2018)	2018	An evaluation algorithm based on Shannon Entropy	–	Percentage of recycling GDP per capita	European Countries
Assessing 28 EU member states' environmental efficiency in national waste generation with DEA (Halkos and Petrou 2019)	2019	VRS CRS DEA models	Labor force Investment Population density Waste	GDP NOx emissions SOx emissions GHG emissions	European Countries
Technology heterogeneity and efficiency of China’s circular economic systems: A game meta-frontier DEA approach (Sun et al. 2019)	2019	Meta-Frontier cooperative game network DEA	*Economic Production*: Fixed asset investments Employed population Total water supply Wastewater *Waste Treatment*: Treatment investment Solid waste disposal. investment	*Economic Production*: GDP Wastewater (Undesirable) Solid waste *Waste Treatment*: Solid waste utilisation Recycled water Wastewater treatment rate	Provinces of China
Ranking European countries on the basis of their environmental and circular economy performance: A DEA application in MSW (Giannakitsidou et al. 2020)	2020	Weight restriction DEA	MSW generated Basic human needs Foundations of wellbeing opportunity	Recycling rate of MSW Circular material use rate	European Countries
Performance measurement for the recycling production system using cooperative game network data envelopment analysis (Huang and Hu 2021)	2021	Cooperative game network DEA	Labor force Capital Energy consumption Energy recovery	GDP per capita Volume of recycled solid waste Volume of backfill Total waste generated	European Countries
The efficiency of circular economies: A comparison of Visegrád Group Countries (Lacko et al. 2021)	2021	VRS CRS DEA models	Waste generated per capita Gross capital formation	Recycling rate of MSW. Circular material use rate	Visegrad Group countries
Assessment of the effectiveness of the European Union countries transition to a circular economy: data envelopment analysis (Temerbulatova et al. 2021)	2021	VRS CRS DEA models	Generation of municipal waste per capita Water exploitation index Final energy consumption Social progress index	Recycling rate of municipal waste Circular material uses rate	European Countries
A design science research methodology for information systems research (Banjerdpaiboon and Limleamthong 2023)	2023	DEA Super-efficiency dual VRS with MPI	Generation of municipal waste per capita Generation of waste excluding major mineral waste per GDP	Recycling rate of municipal waste Recycling rate of packaging waste Recycling biowaste per capita Circular material uses rate	European Countries

Among different DEA models, NDEA models stand out due to their ability to decompose a CE system into interconnected subsystems and account for linkages between them, providing a holistic assessment. Huang and Hu (2021), for example, employed a cooperative game NDEA model to measure performance of CE in the EU countries. It is also important to incorporate undesirable factors, such as waste generation as an output, recycled materials as an undesirable input, are particularly relevant for CE evaluation. In Sect. 5.2, we delve deeper into the challenges of integrating undesirable factors into DEA models, the techniques for addressing these issues, and the implications of randomness in input–output variables in the context of CE efficiency measurement.

## Research framework

To establish a robust methodology for evaluating CE scores across EU countries, our research employs the DSRM approach introduced in Peffers et al. (2007) as outlined in Table 2. The application of DSRM in DEA models has grown significantly in recent years. For instance, Charles et al. (2019) used DSRM to handle dimensionality in DMUs within a DEA framework. Moreover, a DEA-ANN technique was adopted to analyse bank branch performance using DSRM (Tsolas et al. 2020). Santos and Silva (2021) combined DSRM and DEA to optimise IT outsourcing services. Zhu et al. (2022) applied DEA-DSRM to evaluate EU environmental efficiency, focusing on fixed costs and decision objectives. In addition, a DSRM-based technique was created to assess bank branches in DEA models under discrete situations (Omrani et al. 2023). More recently, Arabi et al. (2024) proposed a DEA-TOPSIS model, leveraging DSRM to quantify refrigeration system sustainability.

**Table 2:** DSRM method utilised in this research

DSRM activity	Description	Knowledge base
Problem identification & motivation	Highlight data uncertainties and stochastic factors that complicate CE performance monitoring. Show how existing CE evaluation models neglect random variability, especially in EU datasets	Literature overview on CE performance evaluation frameworks (DEA, NDEA), randomisation issues, and circularity metrics
Define objectives	Develop a reliable, comprehensive CE performance score for EU nations using a strong NDEA-based model and chance-constrained programming to control data uncertainties	Previous CE metrics research indicates stochastic data elements that affect CE assessment accuracy (e.g., circularity investment, waste unpredictability)
Design & development	Develop a CE-specific two-stage NDEA model. Integrate stochastic variables and model economic production and waste treatment as linked subsystems	Chance-constrained programming in DEA, CE NDEA models, and multi-subsystem analysis
Demonstration	Apply the NDEA model to CE data, analysing Economic Production Subsystem (EPS) and Waste Treatment Subsystem (WTS) to determine overall CE efficiency	Empirical data from the Eurostat CE database for 27 EU countries (2013–2020), relevant variables for CE performance measurement, and stochastic DEA model applications
Evaluation	Correlate efficiency ratings to analyse how economic output and waste treatment affect CE performance in each nation. Explore policy implications for enhancing circularity	Evaluation of CE efficiency measures, correlations, and EU sustainability regulations' effects on waste management and circularity
Communication	Explain circularity drivers and suggest EU CE incentives and regulations to stakeholders in sustainability policy and economic modelling	Conclusions from model results, EU CE policy suggestions, and significance to CE performance evaluation and future policy orientations

## CE performance framework and data

This section outlines the key drivers of a CE system, describes the proposed framework, and presents the data along with a randomness test to check data variability.

### CE Framework

A robust CE system is grounded on the 9R principles (e.g., Morseletto 2020; Jacobs et al. 2022; Cong et al. 2017), which advance sustainability by reducing resource depletion and environmental impact while fostering economic growth. These principles prioritise minimising waste and consumption, extending product lifecycles through reuse, repair, refurbishment, remanufacturing and repurposing, and implementing effective recycling and waste management. By adopting CE principles, businesses and societies can also create multifaceted value. Economically, these principles can, for example, drive innovations, open new markets, and create business opportunities. Environmentally, they reduce resource extraction and waste generation, mitigate emissions. Socially, CE drives job creation and encourages sustainable lifestyles. Our research focuses on assessing circularity within production systems, with particular attention to the key drivers of *reduction*, *removal*, and *recycling*.

**Fig. 1:**
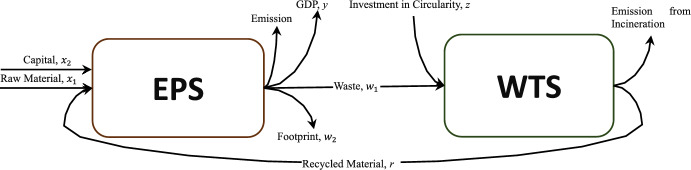
Conceptual network model of CE

**Fig. 2:**
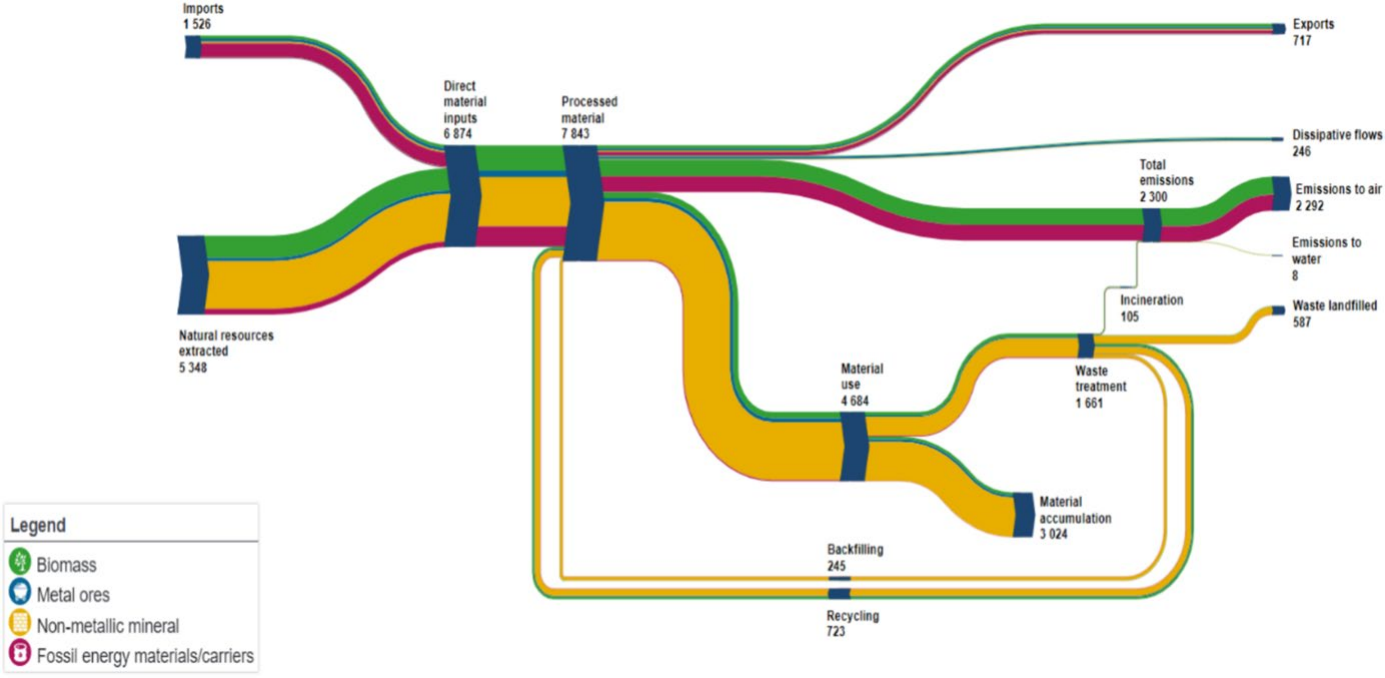
EU year 2020 material flow diagram (https://ec.europa.eu/eurostat/cache/sankey/circular_economy)

Figure 1 conceptualises circularity as two interlinked subsystems: the *Economic Production System* and the *Waste Treatment System*. The EPS captures production lifecycle activities by transforming resources into economic outputs, while the WTS emphasises waste management to minimise environmental harm, maximise resource recovery, and support economic and social goals. Together, these components illustrate the dynamic interplay between production and waste management in country’s material flow, where direct materials are processed into recycled materials (backfilling), emissions, environmental footprints, and waste (see Fig. 2). The evaluation framework integrates economic, environmental, and social dimensions, underscoring the critical interdependence of the EPS and WTS in advancing sustainable development.

In this study, the EPS evaluates economic performance and sustainability through three key inputs: *Raw Material Consumption (RMC), Capital (Cap),* and *Recycled Material (RM). RMC* quantifies the total amount of material extracted (domestically and internationally) for production. *Cap* reflects financial flows, acquisitions and disposals of non-produced non-financial assets, and capital transfers. *RM* measures the extent to which secondary materials are reintegrated into production processes, serving as a key indicator of circularity progress that reduces reliance on primary materials and minimises environmental footprints. The primary desirable output of the EPS is *Gross Domestic Product* (*GDP*) (Jackson 2016; Stern 2004), accompanied by undesirable outputs arising from production and consumption activities such as *General Wastes (GW)* and *Emissions,* which serves as proxies for the environmental impacts of industrial and economic activities*.* For instance, GW accounts for the total volume of waste produced within the country, including major mineral wastes.^1^ A more efficient EPS reduces waste generation through enhanced resource utilisation and sustainable practices, while an effective WTS facilitates the recovery and reintegration of waste into the EPS as secondary materials, thereby closing the circular loop.

Additionally, the system recognises that some waste undergoes energy recovery through incineration, contributing to energy generation but also produces emissions. Another critical metric is the *Footprint,* which captures broader environmental impacts associated with consumption patterns, including both domestic and transboundary effects of imported goods. While rising consumption intensities and shifting consumption patterns drive an increasing consumption footprint, CE initiatives have the potential to reverse these trends by encouraging behavioural changes and improving the environmental performance of products.

The WTS enhances circularity by optimising resource recovery and minimising environmental impact, reframing *waste* streams as opportunities to recover materials and energy. This reduces reliance on raw materials, mitigates environmental degradation, and supports the transition toward a CE. To advance CE practices, significant investments (INV)^2^ are required in key areas such as developing CE infrastructure development, adopting innovative technologies, and implementing advanced waste management systems. These investments are aimed at strengthening a nation’s capacity to efficiently capture, sort, process and manage waste. By producing high-quality secondary resources that can be reintroduced into the EPS, such efforts reduce the need for primary raw materials extraction and promote more sustainable production processes.

### Data

In this study, data was sourced from the CE section of the EUROSTAT database, covering the years 2013 to 2020. This period provides the most comprehensive dataset available for all the variables required in our analysis. Note that correlation tests revealed strong positive correlations between generated wastes, waste disposal, and emissions from sources excluding incineration. Therefore, we exclude waste disposal and emissions variables from the model. In addition, as emissions from incineration constituted a negligible proportion of total emissions and generated waste, they were also omitted from the analysis. Finally, as the focus of this study is the circulation of materials rather than gases, and these factors contribute little to efficiency measures or decision making, we chose not to include them in the models.

### Descriptive statistics

**Fig. 3:**
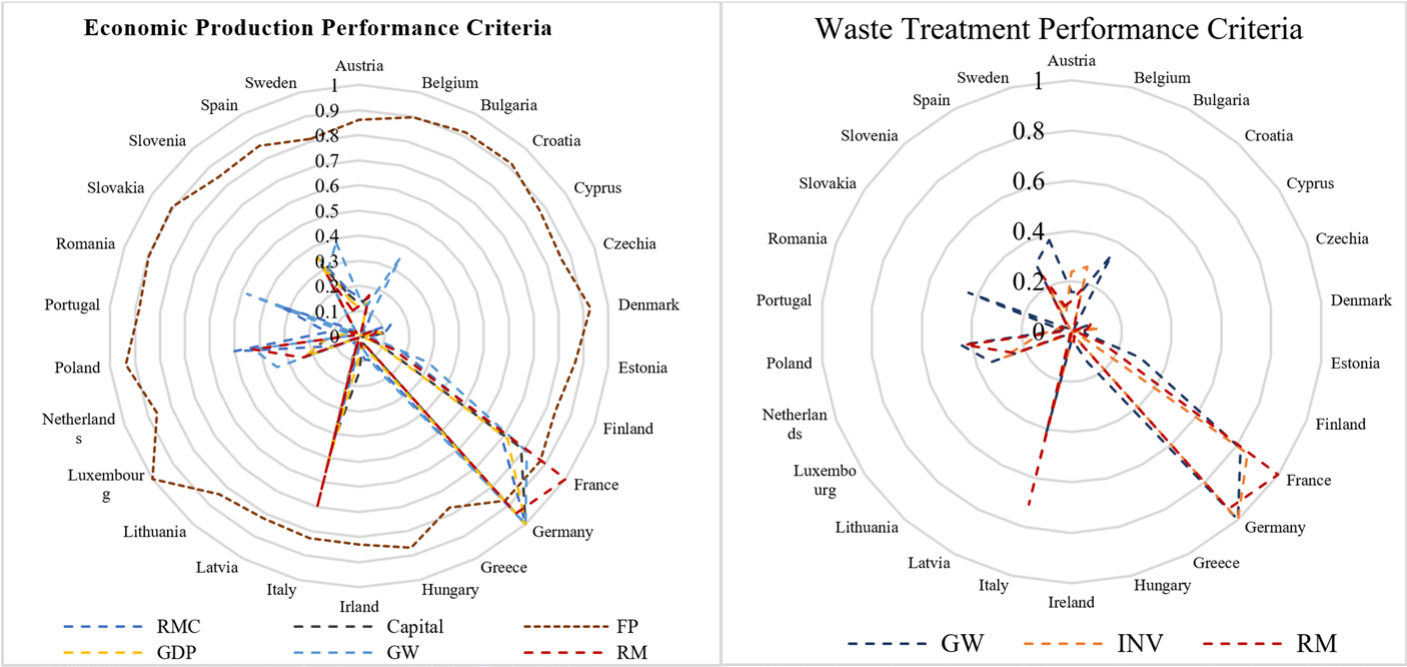
Average EU countries CE performance inputs and outputs in 2013–2020

Using the data summarised in Table 3, we generated diagrams displaying the average magnitudes of the input–output listed for each EU country over the period from 2013 to 2020–see Fig. 3. These diagrams provide a comparative view of each input/output, for example RMC represented as the yearly average, offering insights into the CE performance across EU countries. We find that Germany, France, and Italy emerged as leaders in multiple CE metrics among EU countries, while Luxembourg exhibited a disproportionately high environmental footprint suggesting a need to address consumption patterns and sustainability efforts despite its achievements in other areas.

**Table 3:** Input–output description

Sub-system	Type	Description	Notation	Definition	Unit	Database
EPS	Input	Raw Material Consumption (RMC)	*x*_*1*_	The global demand for the extraction of materials induced by consumption of material within a country minus RM	Kilo Tonnes (kt)	EUROSTAT CE section
		Capital (Cap)	*x*_*2*_	Gross capital of a country minus Investments	Million Dollar (m$)	
		Recycled Material (RM)	*r*	Share of material recovered and fed back into the economy in overall material use times RMC	kt	
	Undesirable Outputs	Generated Waste (GW)	*w*_1_	Total waste generated in a country including major mineral wastes	Million Tonnes (mt)	
		Footprint (FP), Index	*w*_2_	Estimates the environmental impacts of consumption by combining data on consumption intensity and environmental impacts of representative products	na	
	Output	GDP, m$	*y*	Gross Domestic Product	m$	
WTS	Input	Investment in Circularity (INV)	*z*	Gross investment in tangible goods	m€	EUROSTAT CE section
		Generated Waste (GW)	*w*_1_	Total waste generated in a country including major mineral wastes	mt	
	Output	Recycled Material (RM)	*r*	Share of material recovered and fed back into the economy in overall material use times RMC	kt	

These visualisations offer a clear view of each country’s performance across inputs and outputs, enabling the identification of both leaders and areas requiring attention. By examining these patterns, we gain insights into how EU countries prioritise and address key aspects of the CE, such as material efficiency, recycling, and waste management.

Furthermore, we report descriptive statistics for the seven variables included in the study in Table 4. These statistics provide an overview of central tendencies (mean) and variability (value inside the parentheses of the variables), offering insight into the scale and dispersion of the data across EU countries.

**Table 4:** Descriptive statistics of the random variables by the countries in 2013–2020

Country	GDP (× 10^6^)	RMC(× 10^6^)	Cap (× 10^6^)	RM (× 10^6^)	GW (× 10^6^)	INV	FP
Austria	1.17 (0.27)	0.20 (0.05)	0.31 (0.27)	51.84 (0.21)	56.68 (0.13)	5420.44 (0.04)	3.30 (0.03)
Belgium	0.48 (0.12)	0.16 (0.05)	0.11 (0.15)	39.75 (0.22)	59.26 (0.11)	6224.33 (0.19)	5.87 (0.07)
Bulgaria	0.05 (0.27)	0.13 (0.10)	0.01 (0.29)	150.51 (0.19)	156.28 (0.18)	331.22 (0.25)	1.77 (0.09)
Croatia	0.06 (0.14)	0.05 (0.05)	0.01 (0.21)	3.51 (0.19)	4.91 (0.27)	327.22 (0.20)	1.34 (0.11)
Cyprus	0.02 (0.14)	0.02 (0.13)	0.00 (0.29)	1.70 (0.23)	2.06 (0.18)	69.00 (0.38)	0.37 (0.05)
Czechia	0.20 (0.21)	0.17 (0.07)	0.05 (0.20)	22.99 (0.30)	27.94 (0.22)	702.89 (0.24)	3.44 (0.04)
Denmark	0.32 (0.11)	0.13 (0.08)	0.07 (0.15)	15.85 (0.14)	17.64 (0.18)	2673.78 (0.11)	3.10 (0.08)
Estonia	0.02 (0.27)	0.03 (0.09)	0.01 (0.27)	18.52 (0.13)	20.65 (0.12)	161.33 (0.21)	0.45 (0.05)
Finland	0.25 (0.12)	0.25 (0.07)	0.06 (0.14)	95.56 (0.24)	98.12 (0.22)	727.78 (0.07)	2.19 (0.02)
France	2.61 (0.10)	0.89 (0.04)	0.59 (0.12)	307.45 (0.05)	328.25 (0.06)	19,561.89 (0.05)	25.99 (0.02)
Germany	3.52 (0.12)	1.30 (0.02)	0.71 (0.14)	366.43 (0.04)	380.71 (0.05)	23,797.33 (0.37)	35.59 (0.04)
Greece	0.25 (0.21)	0.15 (0.11)	0.04 (0.55)	54.25 (0.37)	56.86 (0.31)	315.44 (0.42)	3.30 (0.05)
Hungary	0.14 (0.15)	0.12 (0.20)	0.03 (0.22)	14.70 (0.20)	18.34 (0.17)	909.56 (0.25)	2.65 (0.04)
Ireland	0.28 (0.28)	0.06 (0.11)	0.08 (0.56)	13.64 (0.35)	18.86 (0.30)	1040.22 (0.85)	2.69 (0.05)
Italy	2.05 (0.09)	0.68 (0.05)	0.39 (0.14)	129.33 (0.11)	161.80 (0.08)	10,118.78 (0.33)	24.84 (0.06)
Latvia	0.03 (0.25)	0.03 (0.08)	0.01 (0.31)	1.53 (0.27)	1.95 (0.28)	207.44 (0.30)	0.84 (0.04)
Lithuania	0.04 (0.26)	0.05 (0.10)	0.01 (0.29)	4.73 (0.09)	6.40 (0.08)	331.44 (0.33)	1.13 (0.03)
Luxembourg	0.06 (0.22)	0.02 (0.07)	0.01 (0.19)	10.54 (0.11)	8.94 (0.11)	557.00 (0.29)	0.30 (0.07)
Netherlands	0.84 (0.11)	0.14 (0.08)	0.17 (0.14)	116.07 (0.18)	120.04 (0.15)	6842.00 (0.26)	9.24 (0.07)
Poland	0.48 (0.24)	0.65 (0.07)	0.09 (0.25)	154.23 (0.11)	162.00 (0.10)	3214.00 (0.27)	13.50 (0.07)
Portugal	0.22 (0.10)	0.17 (0.06)	0.04 (0.20)	13.73 (0.44)	18.86 (0.41)	1625.22 (0.23)	3.90 (0.04)
Romania	0.18 (0.31)	0.44 (0.18)	0.05 (0.37)	184.98 (0.18)	228.02 (0.34)	1000.11 (0.19)	6.37 (0.05)
Slovakia	0.09 (0.18)	0.08 (0.05)	0.02 (0.16)	9.19 (0.23)	11.01 (0.18)	444.44 (0.13)	1.49 (0.11)
Slovenia	0.05 (0.15)	0.03 (0.05)	0.01 (0.24)	5.65 (0.23)	5.92 (0.21)	134.89 (0.44)	0.72 (0.05)
Spain	1.33 (0.12)	0.46 (0.07)	0.30 (0.25)	119.72 (0.16)	134.43 (0.15)	4929.22 (0.18)	22.06 (0.06)
Sweden	0.51 (0.13)	0.25 (0.07)	0.12 (0.16)	121.91 (0.25)	127.33 (0.24)	2024.11 (0.14)	4.46 (0.05)

Our analysis shows that Germany consistently ranks highest across all variables, with France following closely in second place for nearly every indicator. On the other hand, Croatia, Cyprus, Latvia, Lithuania, Luxembourg, and Slovenia have the lowest values for all variables. The coefficient of variation further highlight heterogeneity across countries: Ireland has the highest variation of GDP and INV, Romania has the most variation of RMC and Cap, and similarly, Portugal has the highest variation of RM and GW.

### Randomness of criteria

In this study, we employed spectral analysis using the Fourier transformation to investigate the randomness of our dataset. The Fourier transform decomposes a time series $x\left( t \right)$ into its constituent frequencies, represented mathematically as:

$$X\left( f \right) = \mathop \smallint \limits_{ - \infty }^{ + \infty } x\left( t \right)e^{ - 2\pi ift} dt$$

where $X\left( f \right)$ denotes the Fourier coefficients at frequency *f* (Bingham et al. 1967). By analysing the power spectrum, defined as $P\left( f \right) = \left| {X\left( f \right)} \right|^{2}$, we can identify dominant frequencies and assess the uniformity of power distribution across these frequencies. The null hypothesis for the spectral analysis can be stated as:

**H0:** *The data is random (exhibits a uniform power distribution across frequencies).*

**H1:** *The data is not random (does not exhibit a uniform power distribution across frequencies).*

A Chi-Squared test is then applied to compare the observed frequency distribution with the expected uniform distribution. The p-value obtained from this test can determine whether the null hypothesis is rejected or accepted. A low *p*-value (< 1%) indicates that the data exhibits significant periodicity or structure, suggesting it is not random. This approach is particularly useful in cases where the sample size is limited, as it leverages the inherent properties of the Fourier transform to reveal underlying patterns that may not be apparent through conventional statistical methods.

Table 5 presents the *p*-values of the spectral analysis for each variable across the 26 EU countries. The results show that all seven variables and across all countries, the null hypothesis cannot be rejected, implying that the data follow a random pattern.

**Table 5:** *P*-values of spectral analysis

Country	GDP	RMC	Cap	RM	GW	INV	FP
Austria	0.9768	0.9957	0.9683	0.8856	0.9597	0.9669	0.9370
Belgium	0.9686	0.9922	0.9628	0.966	0.9668	0.9741	0.8334
Bulgaria	0.9673	0.9245	0.982	0.9007	0.9432	0.9892	0.8217
Croatia	0.9928	0.9847	0.9906	0.916	0.9691	0.976	0.7636
Cyprus	0.9914	0.9499	0.9611	0.9224	0.9632	0.9856	0.7720
Czechia	0.9879	0.9798	0.9609	0.8877	0.9496	0.9664	0.8905
Denmark	0.9798	0.9921	0.9919	0.92	0.9581	0.992	0.8633
Estonia	0.9693	0.9882	0.9816	0.9493	0.9391	0.9815	0.8370
Finland	0.9896	0.9668	0.9774	0.9689	0.943	0.9772	0.8125
France	0.9648	0.9609	0.9827	0.9415	0.9568	0.9981	0.8656
Germany	0.9922	0.9281	0.9607	0.912	0.9454	0.9977	0.9549
Greece	0.9959	0.9894	0.9863	0.9484	0.9671	0.9817	0.7791
Hungary	0.9732	0.9463	0.9634	0.9456	0.9453	0.9946	0.7739
Ireland	0.9719	0.9482	0.9642	0.9635	0.942	0.9993	0.8370
Italy	0.9873	0.9591	0.9708	0.9572	0.9565	0.9669	0.8525
Latvia	0.9764	0.9368	0.9577	0.9186	0.9506	0.9835	0.8726
Lithuania	0.9568	0.931	0.9856	0.902	0.9391	0.9916	0.8111
Luxembourg	0.9832	0.972	0.9845	0.9338	0.9394	0.9671	0.8933
Netherlands	0.9975	0.9777	0.9977	0.9511	0.9618	0.9769	0.7730
Poland	0.9573	0.9904	0.9959	0.9353	0.9507	0.9796	0.9765
Portugal	0.9819	0.9962	0.9932	0.8848	0.9488	0.9793	0.9658
Romania	0.9588	0.9645	0.9872	0.9476	0.9509	0.9706	0.9477
Slovakia	0.9839	0.9207	0.9714	0.9664	0.9584	0.9707	0.8611
Slovenia	0.9696	0.9916	0.9756	0.919	0.9468	0.9732	0.8656
Spain	0.9725	0.9209	0.9893	0.9138	0.9542	0.9911	0.8826
Sweden	0.9608	0.9565	0.9708	0.9522	0.9706	0.9907	0.9694

### Normality Criteria

To complement our analysis, we employed the Shapiro–Wilk test to assess the normality of the dataset (Shapiro and Wilk 1965).

Given the limited number of time points, the Shapiro–Wilk test serves as a robust method to evaluate whether the data follows a normal distribution (Souza et al. 2023), which can be formally expressed as:

**H0:** *The data is normally distributed.*

**H1:** *The data is not normally distributed.*

Table 6 reports the *p*-value of the Shapiro–Wilk test. Low *p*-value (< 0.01) are marked with an asterisk (*) to indicate non-normality.

**Table 6:** *P*-values of Shapiro–Wilk test

Country	GDP	RMC	Cap	RM	GW	INV	FP
Austria	0.0031*	0.0032*	0.0059*	0.5267	0.7715	0.4813	0.7038
Belgium	0.0083*	0.0881	0.0123	0.2306	0.4122	0.2087	0.3935
Bulgaria	0.0433	0.2908	0.0254	0.0109	0.0052*	0.3122	0.2351
Croatia	0.0123	0.0002*	0.1018	0.3651	0.2407	0.5602	0.1014
Cyprus	0.0072*	0.2036	0.0583	0.4352	0.0418	0.3881	0.2113
Czechia	0.0208	0.3938	0.0476	0.4370	0.9311	0.1381	0.5098
Denmark	0.0048*	0.9382	0.0267	0.5957	0.2803	0.8762	0.8180
Estonia	0.0501	0.4654	0.0681	0.0270	0.0346	0.0921	0.2894
Finland	0.0007*	0.0251	0.0026*	0.5064	0.5562	0.2063	0.0719
France	0.0009*	0.0198	0.0051	0.6080	0.5509	0.0124	0.2669
Germany	0.0594	0.9338	0.5373	0.9223	0.6056	0.0322	0.7552
Greece	0.1212	0.0242	0.0002*	0.4952	0.7305	0.0803	0.1233
Hungary	0.0238	0.7892	0.1057	0.3979	0.1755	0.1210	0.3460
Ireland	0.0390	0.0006*	0.0020*	0.5759	0.5710	0.0009*	0.2700
Italy	0.0021*	0.0362	0.1253	0.1048	0.5263	0.2800	0.8748
Latvia	0.0162	0.4895	0.1369	0.7856	0.8780	0.2023	0.0462
Lithuania	0.0835	0.6346	0.1478	0.7250	0.0052*	0.4652	0.9509
Luxembourg	0.0106	0.5071	0.0290	0.7223	0.5927	0.5813	0.9673
Netherlands	0.0083*	0.4675	0.0351	0.6095	0.7220	0.4758	0.8901
Poland	0.0321	0.2412	0.0085*	0.8374	0.4378	0.3081	0.0321
Portugal	0.0037*	0.0162	0.0942	0.0007*	0.0018*	0.4447	0.1326
Romania	0.0180	0.8438	0.0207	0.8473	0.0606	0.6037	0.7481
Slovakia	0.0040*	0.0004*	0.0293	0.0699	0.2669	0.4836	0.2144
Slovenia	0.0149	0.0005*	0.1756	0.1810	0.8404	0.0244	0.1629
Spain	0.0007*	0.0059*	0.1842	0.3687	0.6267	0.4357	0.5788
Sweden	0.0033*	0.9981	0.0177	0.3711	0.2786	0.5512	0.0906

The results reveal that GDP consistently displays non-normality at the 1% significance level for about half of the countries. In contrast, RMC displays non-normality in roughly one-quarter of the countries, and CAP in about one-fifth. RM and INV indicate non-normality only in Portugal and Ireland, respectively. Notably, FP data appears normally distributed across all countries. Therefore, these findings highlight variability in the normality of all variables except GDP in our study.

## Methodology

This section introduces the NDEA model developed for evaluating the circularity of material flows at the country level. Subsequently, we apply chance-constrained programming principles to develop a stochastic NDEA model tailored to measure CE performance among EU countries. Our approach provides a robust tool for assessing sustainability outcomes at a national macro-level.

### Different drivers of CE

This research assesses circularity with an emphasis on three key drivers of *reduction*, *removal*, and *recycling* within production systems. To achieve this, we employ a series of DEA models that are tailored to assess CE performance. These models are designed to account for minimisation of CAP, RMC, FP and GW as inputs and undesirable outputs, while enabling the maximisation of undesirable inputs and desirable outputs, such as RM and GDP, within the system. This approach aligns with the overarching objective of enhancing circularity by maximising *reduction* of material input, *removal* of wastes and footprints efficiently, and boosting the *recycling* rates of generated waste. By focusing on these drivers, our framework effectively evaluates and enhances the system’s overall circularity performance.

### Stochastic NDEA models for circular economy performance measurement

This study requires DEA models capable of accounting for undesirable factors (Hatami-Marbini et al. 2022). Among the prominent options, the DDF (Lozano et al. 2024), Slacks-Based Measure (SBM), and Range Adjusted Measure (RAM) are noteworthy, each offering distinct strengths and limitations. The SBM, introduced by Tone (2001), effectively handles input and output slacks but lacks the flexibility for direction-specific efficiency improvements which are essential in sustainability-focused models. The RAM, developed in Cooper et al. (1999), allows for simultaneous adjustments of multiple inefficiencies but is sensitive to outliers and struggles with the dynamic and complex environmental interactions typical in sustainability contexts (Chiu et al. 2014). In contrast, the DDF, introduced by Chung et al. (1997), excels at simultaneously expanding desirable outputs and contracting undesirable ones, aligning closely with the efficiency goals of a CE. Furthermore, DDF’s adaptability to varying disposability conditions makes it the ideal choice for this study (Färe and Grosskopf 2010).

Building upon the DDF framework, we introduce a novel DDF-NDEA model tailored for CE efficiency assessments, particularly in circular supply chain contexts. This model focuses on reducing materials and capital costs while minimising waste generation. Concurrently, it seeks to augment the utilisation of recycled materials derived from waste and to improve overall value addition.

#### Preliminary definitions

Let $\boldsymbol{x}_{n}$ and $\boldsymbol{y}_{n}$ be input and output vector’s corresponding to $\mathrm{DMU}_{n} ,$ respectively. Consider $\boldsymbol{x}$ as input vector of a firm and $\boldsymbol{y}$ as its output vector. The production possibility set can be defined as $P(\boldsymbol{x})=\{(\boldsymbol{x},\boldsymbol{y})\mid \boldsymbol{x}\;\text{can produce}\;\boldsymbol{y}\}$ . Also, let $\boldsymbol{y}_{n}^{U}$ undesirable output vector corresponding to${\mathrm{DMU}}_{n}$. We employ the concept of weak disposability as below:                            

**Weak Disposability**: acknowledges that decreasing undesirable inputs or outputs might be costly. It depicts a more grounded situation in which the mitigation of undesirable factors is linked to technological, financial, or legal limitations. For example, cutting emissions may need spending money on new, expensive technology or procedures. Therefore, weak disposability can be written as $P\left( \boldsymbol{x} \right) = \left\{ {\left( {\boldsymbol{\boldsymbol{x}},\boldsymbol{y}^{U} } \right):\boldsymbol{y} \le \mathop \sum \limits_{n = 1}^{N} \boldsymbol{y}_{n} \lambda_{n} ;\boldsymbol{y}^{U} = \mathop \sum \limits_{n = 1}^{N} \boldsymbol{y}_{n}^{U} \lambda_{n} ;\boldsymbol{x} \ge \mathop \sum \limits_{n = 1}^{N} \boldsymbol{x}_{n} \lambda_{n} ;\lambda_{n} \ge 0;n = 1, \ldots ,N} \right\}$ $P\left( \boldsymbol{x} \right) = \left\{ {\left( {\boldsymbol{\boldsymbol{x}},\boldsymbol{y}^{U} } \right):\boldsymbol{y} \le \mathop \sum \limits_{n = 1}^{N} \boldsymbol{y}_{n} \lambda_{n} ;\boldsymbol{y}^{U} = \mathop \sum \limits_{n = 1}^{N} \boldsymbol{y}_{n}^{U} \lambda_{n} ;\boldsymbol{x} \ge \mathop \sum \limits_{n = 1}^{N} \boldsymbol{x}_{n} \lambda_{n} ;\lambda_{n} \ge 0;n = 1, \ldots ,N} \right\}$ where $\lambda_{n}$ and $N$ denote intensity variable and the number of DMUs respectively. Additionally, we assume that both desirable and undesirable outputs are produced jointly, a condition known as '*null-jointness*'. This implies that it is impossible to generate desirable outputs without simultaneously producing some undesirable outputs. In other words, a reduction in desirable outputs would necessitate a corresponding reduction in undesirable outputs. In addition, we introduce the definition of circular disposability as below:

**Circular Disposability** is a situation in which a DMU, say $\mathrm{DMU}_{o}$ seeks for solutions to simultaneously increase desirable outputs $\boldsymbol{y}_{o}$ and undesirable inputs $\boldsymbol{x}_{o}^{U}$ along with decreasing undesirable outputs $\boldsymbol{y}_{o}^{U}$ and desirable inputs $\boldsymbol{x}_{o}$. We can formulate the circular disposability as $P\left( {\boldsymbol{x}_{o} ,\boldsymbol{x}_{o}^{U} } \right) = \left\{ {\left( {\boldsymbol{y},\boldsymbol{y}^{U} } \right):\boldsymbol{y} \le \mathop \sum \limits_{n = 1}^{N} \boldsymbol{y}_{n} \lambda_{n} ;\boldsymbol{y}^{U} = \mathop \sum \limits_{n = 1}^{N} \boldsymbol{y}_{n}^{U} \lambda_{n} ;\boldsymbol{x} \ge \mathop \sum \limits_{n = 1}^{N} \boldsymbol{x}_{n} \lambda_{n} ;\boldsymbol{x}^{U} = \mathop \sum \limits_{n = 1}^{N} \boldsymbol{x}_{n}^{U} \lambda_{n} ; \lambda_{n} \ge 0;n = 1, \ldots ,N} \right\}$. In this definition an increase in circular inputs results in enhanced efficiency. It is worth noting that equality in $\boldsymbol{x}^{U} = \textstyle\sum_{n = 1}^{N} \boldsymbol{x}_{n}^{U} \lambda_{n}$, like $\boldsymbol{y}^{U} = \textstyle\sum_{n = 1}^{N} \boldsymbol{y}_{n}^{U} \lambda_{n}$, ensures the *null-jointness* property among desirable and undesirable outputs as well as undesirable inputs.    

#### Deterministic DEA DDF models

Here, we adjust the fundamental DDF model introduced in Chung et al. (1997) to measure the inefficiency of EPS, as formulated in Model (1):

1 $$\begin{gathered} \max \beta \hfill \\ s. \, t. \hfill \\ \mathop \sum \limits_{n = 1}^{N} \lambda_{n} x_{mn} \le x_{mo} - \beta g_{xmo} \quad m \, = \, 1, \, 2\quad (1.1) \hfill \\ \mathop \sum \limits_{n = 1}^{N} \lambda_{n} r_{n} = r_{o} + \beta g_{yso} \quad \quad \qquad \qquad \qquad \; (1.2) \hfill \\ \mathop \sum \limits_{n = 1}^{N} \lambda_{n} y_{n} \ge y_{o} + \beta g_{yo} \quad \qquad \qquad \qquad \quad \; \; (1.3) \hfill \\ \mathop \sum \limits_{n = 1}^{N} \lambda_{n} w_{in} = w_{io} - \beta g_{wio} \quad i = \, 1, \, 2\quad \quad \; \; \left({1.4} \right) \hfill \\ \lambda_{1} , \ldots ,\lambda_{N} \ge 0;\beta \ge 0 \hfill \\ \end{gathered} $$

where $\lambda$ and $\beta$ are intensity and distance to the efficiency frontier of EPS variables respectively. One can find the definitions of $\boldsymbol{x}$, $r$, $y$, and $\boldsymbol{w}$ in Table 3. The objective function of Model (1) is designed to locate the optimal point on the efficient frontier with minimum $\boldsymbol{x}$ and $\boldsymbol{w}$ and maximum $r$ and $y$ to the fullest extent possible. This objective aligns with core CE principles by balancing economic growth with minimised environmental impact which is further discussed in Sects.  4.1 and 5.1.        

Note that constraints (1.1) and (1.3) are designed to maximise the reduction of conventional inputs, RMC and CAP, while increasing the conventional output, GDP, by the level of $\beta$ respectively, Constraint (1.2) treats $r$ as an undesirable input. Here, the objective is to increase the proportion of recycled materials incorporated into production processes. Unlike conventional inputs, which are typically minimised to improve efficiency, undesirable inputs are specific resources or factors whose increase, in conjunction with outputs, is favourable in certain efficiency analyses (Jahanshahloo et al. 2005). This increase may support performance measures in contexts where a greater occurrence of these inputs highlights the effectiveness of interventions. Conversely, Constraint (1.4) pertains to $w_{i}$, indicating the need to minimise GW and FP. The equality conditions in both Constraints (1.2) and (1.4) uphold the null-jointness property, ensuring that any increase in recycled materials aligns with a corresponding decrease in waste inputs. This interplay between undesirable factors, which is represented by grounded in the null-jointness property, has been thoroughly examined in previous studies on CE efficiency (Chung et al. 1997). In Sects.  4.1 and 5.1, we discuss our objective in this study to maximise material reduction, waste and footprint removal, and recycling efforts within the CE framework. To achieve this, we select the direction vector $\boldsymbol{g}_{EPS} = \left( { - \boldsymbol{g}_{x} ,g_{y} , - \boldsymbol{g}_{w} } \right) = \left( { - \boldsymbol{x}_{o} ,y_{o} , - \boldsymbol{w}_{o} } \right)$ which corresponds to the economic production system where $- \boldsymbol{g}_{x}$ represents a reduction in material usage, $g_{y}$ signifies an increase in $y$, and $- \boldsymbol{g}_{w}$ denotes waste removal.     This direction vector indicates our objective to simultaneously minimise $\boldsymbol{x}$ and $\boldsymbol{w}$, while maximising $y$ , thus aligning economic growth with resource efficiency. By choosing this direction, we aim to ensure that our models support the reduction of raw material consumption and capital, minimise waste generation, and facilitate responsible waste disposal, all while contributing to the expansion of GDP.         Similarly, we adjust the fundamental DDF model introduced in Chung et al. (1997) for measuring the efficiency of WTS to formulate the following model (2):

2 $$\begin{gathered} \max \theta \hfill \\ s. \, t. \hfill \\ \mathop \sum \limits_{n = 1}^{N} \mu_{n} w_{in} \le w_{io} - \beta g_{wio} \quad i = 1,2\quad \left( {2.1} \right) \hfill \\ \mathop \sum \limits_{n = 1}^{N} \mu_{n} z_{n} \le z_{o} \qquad \qquad \qquad \qquad \qquad \left( {2.2} \right) \hfill \\ \mathop \sum \limits_{n = 1}^{N} \mu_{n} r_{n} \ge r_{o} + \theta g_{ro} \qquad \qquad \qquad \quad \left( {2.3} \right) \hfill \\ \mu_{1} , \ldots ,\mu_{N} \ge 0;\theta \ge 0 \hfill \\ \end{gathered} $$

where $\mu$ and $\theta$ are intensity, distance to the efficient frontier of WTS variables respectively. One can find the definition of $z$ in Table 3. The objective function in Model (2) is crafted to identify the optimal efficiency point on the frontier by minimising the consumption of $\boldsymbol{w}$ while maximising the production of $r$ to their fullest potential.    

In addition, Constraints (2.1) and (2.3) are designed to maximise the reduction of waste inputs, GW, and to enhance the recycled output, RM, respectively, at the level of θ. Furthermore, an additional constraint. (2.2) is included to ensure that a reduction in INV is not advantageous; as an input to the WTS, higher INV results in a lower efficiency score for a country’s WTS, relative to countries with lower INV.

Analogously, we chose $\boldsymbol{g}_{WTS} = ( - \boldsymbol{g}_{w} ,g_{r} ) = \left( { - \boldsymbol{w},r} \right)$ as the direction vector corresponding to the economic production system. By choosing this direction, we ensure that our model not only promotes the reduction of emissions from incineration but also supports an increase in gross value added through circularity and the use of recycled materials.

#### Deterministic NDEA DDF models

Considering the conceptual model presented in Fig. 1, we develop the following two-stage NDEA model which not only integrates Models (1) and (2) but also imposes two constraints: (i) addressing the paradigm of sharing the waste from EPS to WTS for recycling, (ii) closing the CE loop which observes the feeding of the recycled material from the WTS back into the EPS (3).

3 $$\begin{gathered} \max \delta = \pi \beta + \left( {1 - \pi } \right)\theta \hfill \\ s. \, t. \hfill \\ \mathop \sum \limits_{n = 1}^{N} \lambda_{n} x_{mn} \le \left( {1 - \beta } \right)x_{mo} \quad m = 1,2\qquad \left( {3.1} \right) \hfill \\ \mathop \sum \limits_{n = 1}^{N} \lambda_{n} r_{n} = \left( {1 + \beta } \right)r_{o} \quad \qquad \quad \qquad \qquad \; \left( {3.2} \right) \hfill \\ \mathop \sum \limits_{n = 1}^{N} \lambda_{n} y_{n} \ge \left( {1 + \beta } \right)y_{o} \qquad \qquad \qquad \qquad \; \left( {3.3} \right) \hfill \\ \mathop \sum \limits_{n = 1}^{N} \lambda_{n} w_{in} = \left( {1 - \beta } \right)w_{io} \quad i = 1,2\qquad \quad \left( {3.4} \right) \hfill \\ \mathop \sum \limits_{n = 1}^{N} \mu_{n} w_{1n} \le \left( {1 - \theta } \right)w_{1o} \quad \qquad \qquad \qquad \left( {3.5} \right) \hfill \\ \mathop \sum \limits_{n = 1}^{N} \mu_{n} z_{n} \le z_{0} \qquad \qquad \qquad \qquad \; \; \qquad \quad \left( {3.6} \right) \hfill \\ \mathop \sum \limits_{n = 1}^{N} \mu_{n} r_{n} = \left( {1 + \theta } \right)r_{o} \qquad \qquad \quad \qquad \quad \; \left( {3.7} \right) \hfill \\ \mathop \sum \limits_{n = 1}^{N} \mu_{n} w_{1n} \le \mathop \sum \limits_{n = 1}^{N} \lambda_{n} w_{1n} \qquad \qquad \quad \quad \quad \; \left( {3.8} \right) \hfill \\ \mathop \sum \limits_{n = 1}^{N} \lambda_{n} r_{n} \le \mathop \sum \limits_{n = 1}^{N} \mu_{n} r_{n} \quad \qquad \qquad \qquad \quad \; \; \left( {3.9} \right) \hfill \\ \lambda_{1} , \ldots ,\lambda_{N} \ge 0;\mu_{1} , \ldots ,\mu_{N} \ge 0;\beta ,\theta \ge 0 \hfill \\ \hfill \\ \end{gathered} $$

where $\pi \in \left[ {0,1} \right]$ denotes the priority which is assigned to EPS in a cooperative game model, $\delta$ is a convex combination of $\beta$ and $\theta$, Constraints (3.1–3.4) represent WPS transferred from Model (1), and Constraints (3.5–3.7) denote WTS adopted from Model (2). The equality condition in (3.7) preserves the null-jointness property which is imposed by (1.2) and (1.4) when Models (1) and (2) are combined to form Model (3). It ensures that if no waste is generated, then no recycled material is produced either. Briefly, this convex combination reflects that the total CE efficiency is calculated as a weighted combination of the two subsystems: EPS and WTS. Here, π represents the relative importance assigned to EPS efficiency, while $\left( {1 - \pi } \right)$ denotes the relative importance given to WTS efficiency by researchers or decision-makers. Additionally, Constraint (3.8) establishes the interconnection between GW by the EPS and the waste handled by the WTS, ensuring material flows between these systems. Similarly, Constraint (3.9) defines the relationship between the generated waste treated by the WTS and the RM supplied back to the EPS, effectively creating a closed loop within the model. This loop, supported by the maximisation constraints in Constraint (3.2), ensures that recycled material usage is prioritised, reinforcing the model’s commitment to CE principles. Through this configuration, the model robustly represents the circular flow of materials, aligning with the core CE goal of sustaining material circulation and minimising waste.    

Now, we let Total CE Efficiency = 1/(1 + $\delta$^*^), EPS Efficiency = 1/(1 + β^*^), and WTS Efficiency = 1/(1 + $\theta$^*^). In addition, one can adjust $\pi$ to develop more case-specific scenarios. Here, we introduce the following theorem, which sets out the relationship between EPS and WTS inefficiencies.

##### Theorem 1:

In Model (3) $$\beta^{*} \le \theta^{*}$$.

##### Proof:

From constraints (3.2), (3.7), and (3.9), we obtain $$\left( {1 + \beta } \right)r_{o} $$$$= \textstyle\sum_{n = 1}^{N} \lambda_{n} r_{n} \le \textstyle\sum_{n = 1}^{N} \mu_{n} r_{n}$$$$= \left( {1 + \theta } \right)r_{o}$$, which follows that $$\left( {1 + \beta } \right)r_{o} $$$$\le \left( {1 + \theta } \right)$$ . Dividing both sides by $$r_{o}$$ (with $$r_{o} > 0$$ ) yeils $$\beta \le \theta$$. Therefore, we conclude that $$\beta^{*} \le \theta^{*}$$.□

##### Corollary 1:

If a country is WTS-efficient it is EPS-efficient.

##### Corollary 2:

A country is CE-efficient if and only if it is WTS-efficient.

#### Chance-constrained NDEA DDF models

In this section, by considering random variables ${\widetilde{x}}_{1}$, ${\widetilde{w}}_{1}$ and $\widetilde{r}$, we can formulate the following equivalent model to Model (3) under uncertainty (4):

4 $$\begin{gathered} \max \delta = \pi \beta + \left( {1 - \pi } \right)\theta \hfill \\ s. \, t. \hfill \\ \mathop \sum \limits_{n = 1}^{N} \lambda_{n} \tilde{x}_{1n} \le \left( {1 - \beta } \right)\tilde{x}_{1o} \quad \; \left( {4.1} \right) \hfill \\ \mathop \sum \limits_{n = 1}^{N} \lambda_{n} x_{2n} \le \left( {1 - \beta } \right)x_{2o} \quad \; \left( {4.2} \right) \hfill \\ \mathop \sum \limits_{n = 1}^{N} \lambda_{n} \tilde{r}_{n} = \left( {1 + \beta } \right)\tilde{r}_{o} \quad \quad \; \left( {4.3} \right) \hfill \\ \mathop \sum \limits_{n = 1}^{N} \lambda_{n} y_{n} \ge \left( {1 + \beta } \right)y_{o} \quad \quad \; \left( {4.4} \right) \hfill \\ \mathop \sum \limits_{n = 1}^{N} \lambda_{n} \tilde{w}_{1n} = \left( {1 - \beta } \right)\tilde{w}_{1o} \quad \left( {4.5} \right) \hfill \\ \mathop \sum \limits_{n = 1}^{N} \lambda_{n} w_{2n} = \left( {1 - \beta } \right)w_{2o} \quad \left( {4.6} \right) \hfill \\ \mathop \sum \limits_{n = 1}^{N} \mu_{n} \tilde{w}_{1n} \le \left( {1 - \theta } \right)\tilde{w}_{1o} \quad \left( {4.7} \right) \hfill \\ \mathop \sum \limits_{n = 1}^{N} \mu_{n} z_{n} \le z_{o} \quad \quad \quad \quad \quad \; \; \left( {4.8} \right) \hfill \\ \mathop \sum \limits_{n = 1}^{N} \mu_{n} \tilde{r}_{n} = \left( {1 + \theta } \right)\tilde{r}_{o} \quad \quad \; \left( {4.9} \right) \hfill \\ \mathop \sum \limits_{n = 1}^{N} \mu_{n} \tilde{w}_{1n} \le \mathop \sum \limits_{n = 1}^{N} \lambda_{n} \tilde{w}_{1n} \quad \; \; \left( {4.10} \right) \hfill \\ \mathop \sum \limits_{n = 1}^{N} \lambda_{n} \tilde{r}_{n} \le \mathop \sum \limits_{n = 1}^{N} \mu_{n} \tilde{r}_{n} \quad \quad \; \; \; \left( {4.11} \right) \hfill \\ \lambda_{1} , \ldots ,\lambda_{N} \ge 0;\mu_{1} , \ldots ,\mu_{N} \ge 0;\beta ,\theta \ge 0 \hfill \\ \end{gathered} $$

To address the issue of data randomness, we employ a chance-constrained approach. The method, originally introduced in Charnes and Cooper (1959), serves as a novel conceptual and analytical framework to address challenges in uncertain temporal planning. This involves the exploration of optimal stochastic decision rules. Later, chance-constrained programming was employed as an alternative stochastic approach to DEA in Land et al. (1993). Over the years, this approach has seen significant advancements, with its application expanding across various contexts (Olesen and Petersen 1995; Cooper et al. 1998; Cooper et al. 2004).

This study utilises the chance-constrained programming methodology to present a ground-breaking CE-NDEA model that addresses uncertainties in criteria outcomes. Toward this end, let $$\tilde{w}_{j} = \left( {\tilde{w}_{1j} \ldots \tilde{w}_{Ij} } \right)$$ stand for INV vector. We define $$\tilde{k}_{o} = \textstyle\sum_{n = 1}^{N} \mu_{n} \tilde{w}_{1n} - \left( {1 - \theta } \right)\tilde{w}_{1o}$$ for $$o = 1, \ldots ,N$$ which is normally distributed. Hence, $$P\left\{ {\textstyle\sum\limits_{{n = 1}}^{N} {\mu _{n} } \tilde{w}_{{1n}} - \left( {1 - \theta } \right)\tilde{w}_{{1o}} \le 0} \right\} = P\left\{ {\tilde{k}_{o} \le 0} \right\} $$$$ = P\left\{ {\frac{{\tilde{k}_{o} - E\left\{ {\tilde{k}_{o} } \right\}}}{{\sqrt {V\left\{ {\tilde{k}_{o} } \right\}} }} \le \frac{{ - E\left\{ {\tilde{k}_{o} } \right\}}}{{\sqrt {Var\left\{ {\tilde{k}_{o} } \right\}} }}} \right\}$$. Let $$F$$ be the cumulative distribution function of the standard normal distribution it follows that $$P\left\{ {\tilde{k}_{o} \le 0} \right\} = F\left\{ {\frac{{ - E\left\{ {\tilde{k}_{o} } \right\}}}{{\sqrt {Var\left\{ {\tilde{k}_{o} } \right\}} }}} \right\}$$. Let $$\Phi^{ - 1} \left( \alpha \right)$$ be the standard normal value such that $$F\left( {\Phi^{ - 1} \left( \alpha \right)} \right) = \alpha$$ . Then the statement $$P\left\{ {\tilde{k}_{o} \le 0} \right\}$$ is realised if and only if $$\frac{{ - E\left\{ {\tilde{k}_{o} } \right\}}}{{\sqrt {Var\left\{ {\tilde{k}_{o} } \right\}} }} \ge \Phi^{ - 1} \left( \alpha \right)$$ or equivalently $$E\left\{ {\tilde{k}_{o} } \right\} - \Phi^{ - 1} \left( \alpha \right)\sqrt {Var\left\{ {\tilde{k}_{o} } \right\}} \le 0$$. Note that $$E\left\{ {\tilde{k}_{o} } \right\} = E\left\{ {\textstyle\sum_{n = 1}^{N} \mu_{n} \tilde{w}_{1n} - \left( {1 - \theta } \right)\tilde{w}_{1o} } \right\} = \textstyle\sum_{n = 1}^{N} \mu_{n} w_{1n} - \left( {1 - \theta } \right)w_{1o}$$ and $$Var\left( {\tilde{k}_{o} } \right) = Var\left( {\textstyle\sum_{{n = 1}}^{N} {\mu _{n} } \tilde{w}_{{1n}} - \left( {1 - \theta } \right)\tilde{w}_{{1o}} } \right) = \sqrt {\textstyle\sum_{{n = 1}}^{N} {\mu _{n}^{2} } \left( {\sigma _{{1n}}^{w} } \right)^{2} + \left( {1 - \theta } \right)^{2} \left( {\sigma _{{1o}}^{w} } \right)^{2} } $$$$ \ge \sum\limits_{{n = 1}}^{N} {\mu _{n} } \sigma _{{1n}}^{w} + \left( {1 - \theta } \right)\sigma _{{1o}}^{w} $$. In addition, since $$\frac{{\tilde{k}_{o} - E\left\{ {\tilde{k}_{o} } \right\}}}{{\sqrt {V\left\{ {\tilde{k}_{o} } \right\}} }}$$ follows standard normal distribution from $$ P\left\{ {\frac{{\tilde{k}_{o} - E\left\{ {\tilde{k}_{o} } \right\}}}{{\sqrt {V\left\{ {\tilde{k}_{o} } \right\}} }} \le \frac{{ - E\left\{ {\tilde{k}_{o} } \right\}}}{{\sqrt {Var\left\{ {\tilde{k}_{o} } \right\}} }}} \right\} $$$$ \ge 1 - \alpha $$ we obtain $$\textstyle\sum_{n = 1}^{N} \mu_{n} \left( {w_{1n} - \Phi^{ - 1} \left( \alpha \right)\sigma_{1n}^{w} } \right) - \left( {1 - \theta } \right)\left( {w_{1o} - \Phi^{ - 1} \left( \alpha \right)\sigma_{1o}^{w} } \right) \le 0$$.

Analogously, we can rewrite the other constraints including random variables ${\widetilde{x}}_{1}$ and $\widetilde{r}$ to formulate the following model (5).

5 $$\begin{gathered} \max \delta = \pi \beta + \left( {1 - \pi } \right)\theta \hfill \\ s. \, t. \hfill \\ \mathop \sum \limits_{n = 1}^{N} \lambda_{n} \left( {x_{1n} - \Phi^{ - 1} \left( \alpha \right)\sigma_{1n}^{x} } \right) \le \left( {1 - \beta } \right)(x_{1o} - \Phi^{ - 1} \left( \alpha \right)\sigma_{1o}^{x} )\quad \left( {5.1} \right) \hfill \\ \mathop \sum \limits_{n = 1}^{N} \lambda_{n} x_{2n} \le \left( {1 - \beta } \right)x_{2o} \quad \qquad \qquad \qquad \qquad \qquad \; \qquad \qquad \; (5.2) \hfill \\ \mathop \sum \limits_{n = 1}^{N} \lambda_{n} \left( {r_{n} - \Phi^{ - 1} \left( \alpha \right)\sigma_{n}^{r} } \right) = \left( {1 + \beta } \right)(r_{o} - \Phi^{ - 1} \left( \alpha \right)\sigma_{o}^{r} )\quad \qquad (5.3) \hfill \\ \mathop \sum \limits_{n = 1}^{N} \lambda_{n} y_{n} \ge \left( {1 + \beta } \right)y_{o} \quad \qquad \qquad \qquad \qquad \quad \quad \qquad \qquad \quad \; \; (5.4) \hfill \\ \mathop \sum \limits_{n = 1}^{N} \lambda_{n} \left( {w_{1n} - \Phi^{ - 1} \left( \alpha \right)\sigma_{1n}^{w} } \right) = \left( {1 - \beta } \right)(w_{1o} - \Phi^{ - 1} \left( \alpha \right)\sigma_{1o}^{w} )\quad (5.5) \hfill \\ \mathop \sum \limits_{n = 1}^{N} \lambda_{n} w_{2n} = \left( {1 - \beta } \right)w_{2o} \quad \qquad \qquad \qquad \qquad \qquad \qquad \qquad \; (5.6) \hfill \\ \mathop \sum \limits_{n = 1}^{N} \mu_{n} \left( {w_{1n} - \Phi^{ - 1} \left( \alpha \right)\sigma_{1n}^{w} } \right) \le \left( {1 - \theta } \right)(w_{1o} - \Phi^{ - 1} \left( \alpha \right)\sigma_{1o}^{w} )\quad (5.7) \hfill \\ \mathop \sum \limits_{n = 1}^{N} \mu_{n} z_{n} \le z_{o} \quad \qquad \qquad \qquad \qquad \qquad \quad \qquad \qquad \qquad \quad \; \; \; (5.8) \hfill \\ \mathop \sum \limits_{n = 1}^{N} \lambda_{n} \mu_{n} \left( {r_{n} - \Phi^{ - 1} \left( \alpha \right)\sigma_{n}^{r} } \right) = \left( {1 + \theta } \right)(r_{o} - \Phi^{ - 1} \left( \alpha \right)\sigma_{o}^{r} )\quad \quad (5.9) \hfill \\ \mathop \sum \limits_{n = 1}^{N} \left( {\mu_{n} - \lambda_{n} } \right)\left( {w_{1n} - \Phi^{ - 1} \left( \alpha \right)\sigma_{1n}^{w} } \right) \le 0\quad \qquad \qquad \qquad \qquad \; (5.10) \hfill \\ \mathop \sum \limits_{n = 1}^{N} \left( {\lambda_{n} - \mu_{n} } \right)\left( {r_{n} - \Phi^{ - 1} \left( \alpha \right)\sigma_{n}^{r} } \right) \le 0\quad \qquad \qquad \qquad \qquad \quad \; (5.11) \hfill \\ \lambda_{1} , \ldots ,\lambda_{N} \ge 0;\mu_{1} , \ldots ,\mu_{N} \ge 0;\beta ,\theta \ge 0 \hfill \\ \end{gathered} $$

##### Remark 1:

Theorem 1, Corollaries 1 and 2 are readily applicable to Model (4) and Model (5).

**Fig. 4:**
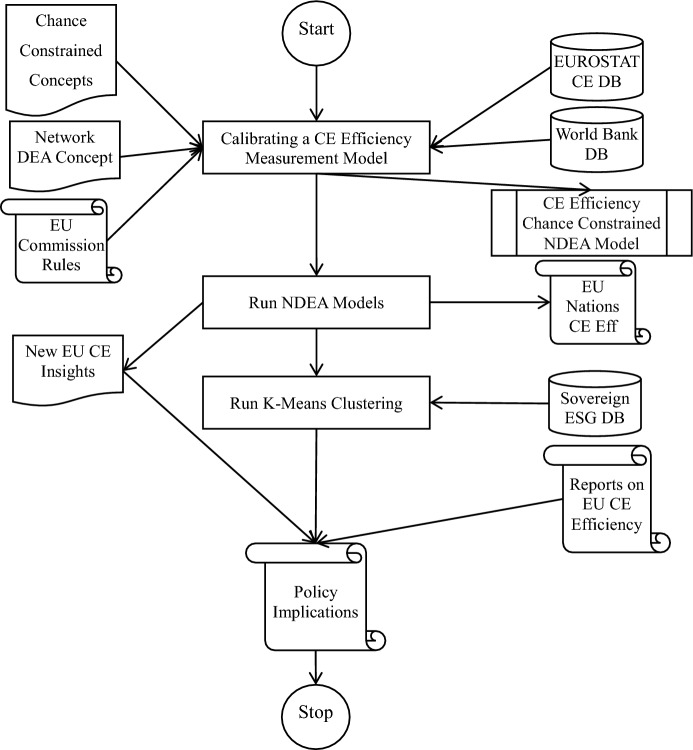
Chance-constrained NDEA CE efficiency measurement flowchart

This study employs Model (5) as the primary tool for evaluating the CE of EU countries. To provide a clear overview of our research process, we present a flowchart in Fig. 4 to highlight the key steps of our studies and assist future researchers in replicating our study or adapting our models.

Figure 4 presents a structured flowchart of the chance-constrained NDEA model-based CE efficiency measurement procedure. Calibrating the CE efficiency assessment model uses network DEA and chance-constrained programming.

These theoretical underpinnings drive the creation of a two-stage model linking the EPS and WTS with stochastic aspects to solve CE data uncertainties. Next, data for the model are sourced primarily from EUROSTAT (for CE-related measures) and the World Bank (for macroeconomic indicators), ensuring a rigorous and comprehensive foundation. After operationalisation, the model evaluates EU nations' CE performance, generating subsystem-level and overall efficiency ratings. Use K-means clustering to detect trends and categorise nations by cyclical performance. Combining model outputs with sovereign ESG data and benchmarking against EU Commission laws and recommendations contextualises the conclusions. This multidimensional research identifies CE implementation strengths, limitations, and policy needs in each nation. Policy implications and strategic insights to support EU-wide circularity activities are the last phases.

## Results and analysis

This section presents the key findings from our proposed model. Following this, we delve into the foundational aspect of NDEA, its capacity to decompose the total system efficiency into EPS and WTS subsystems.

### CE rankings

We executed our models using AIMMS v-24.5.9.4- × 64, applying parameter settings that balance the priorities between subsystems. To be more specific, we set $\pi = 0.5$ in Model (5) to allocate equal importance to EPS and WTS in a country’s CE framework. Additionally, $\alpha = 0.2$ was selected, corresponding to $\Phi^{ - 1} \left( \alpha \right) = - 0.84$ as obtained from a cumulative normal distribution table.

**Fig. 5:**
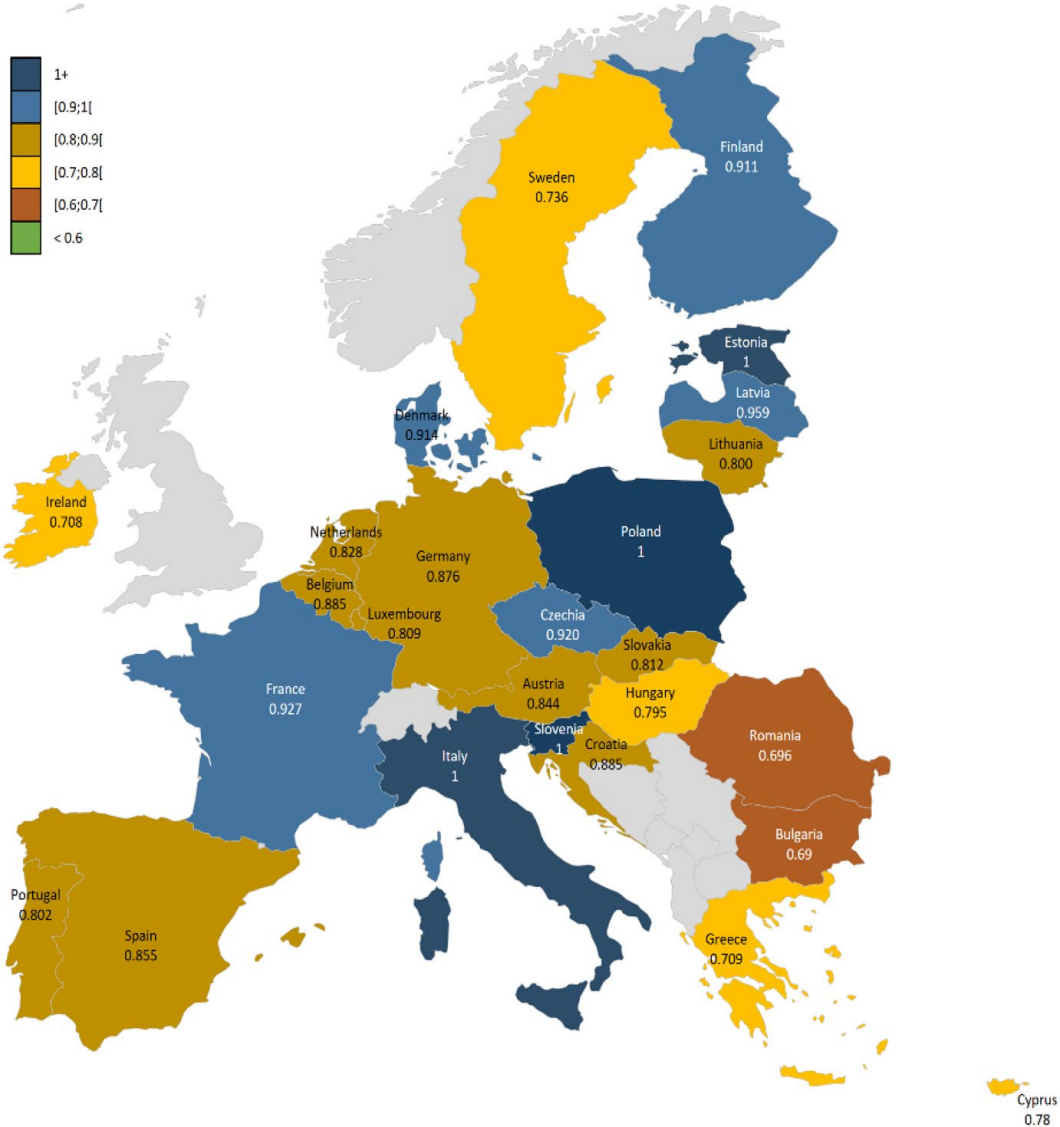
Total CE efficiencies of EU countries

Figure 5 and Table 7 presents the results, sorted by total efficiency scores, with equal weighting assigned to EPS and WTS. The results provide important insights into the dynamics between subsystem performances. We find that any country achieving WTS efficiency also attains total CE efficiency, highlighting the pivotal importance of waste management systems in determining overall CE performance. However, EPS efficiency alone does not guarantee CE efficiency. For example, the Austria is identified as EPS-efficient but WTS-inefficient, which results in it being total CE-inefficient.

**Table 7:** CE efficiencies of EU countries

Country	Total efficiency	Economic production	Waste treatment efficiency	Country	Total efficiency	Economic production	Waste treatment efficiency
Estonia	1.0000	1.0000	1.0000	Austria	0.8439	1.0000	0.7299
Italy	1.0000	1.0000	1.0000	Netherlands	0.8290	1.0000	0.7079
Poland	1.0000	1.0000	1.0000	Hungary	0.8251	0.8567	0.7958
Slovenia	1.0000	1.0000	1.0000	Slovakia	0.8121	0.8931	0.7446
Latvia	0.9585	1.0000	0.9203	Luxembourg	0.8094	1.0000	0.6799
France	0.9269	1.0000	0.8638	Portugal	0.8018	1.0000	0.6691
Czechia	0.9196	0.9196	0.9196	Lithuania	0.8004	0.9015	0.7198
Denmark	0.9138	0.9988	0.8421	Cyprus	0.7803	1.0000	0.6398
Finland	0.9111	0.9111	0.9111	Sweden	0.7358	1.0000	0.5821
Belgium	0.8853	0.9681	0.8156	Greece	0.7091	1.0000	0.5493
Croatia	0.8847	0.9584	0.8215	Ireland	0.7079	1.0000	0.5479
Germany	0.8761	1.0000	0.7796	Romania	0.6961	1.0000	0.5339
Spain	0.8551	1.0000	0.7469	Bulgaria	0.6898	1.0000	0.5265

However, the findings suggest that further improvements in WTS are necessary to fully realise its CE potential an achieve total efficiency. This analysis underscores the necessity of balanced performance across both subsystems to achieve optimal CE outcomes. These results are consistent with Theorem 1 and Corollaries 1 and 2.

### Pairwise Spearman’s correlation coefficients of efficiency scores

**Fig. 6:**
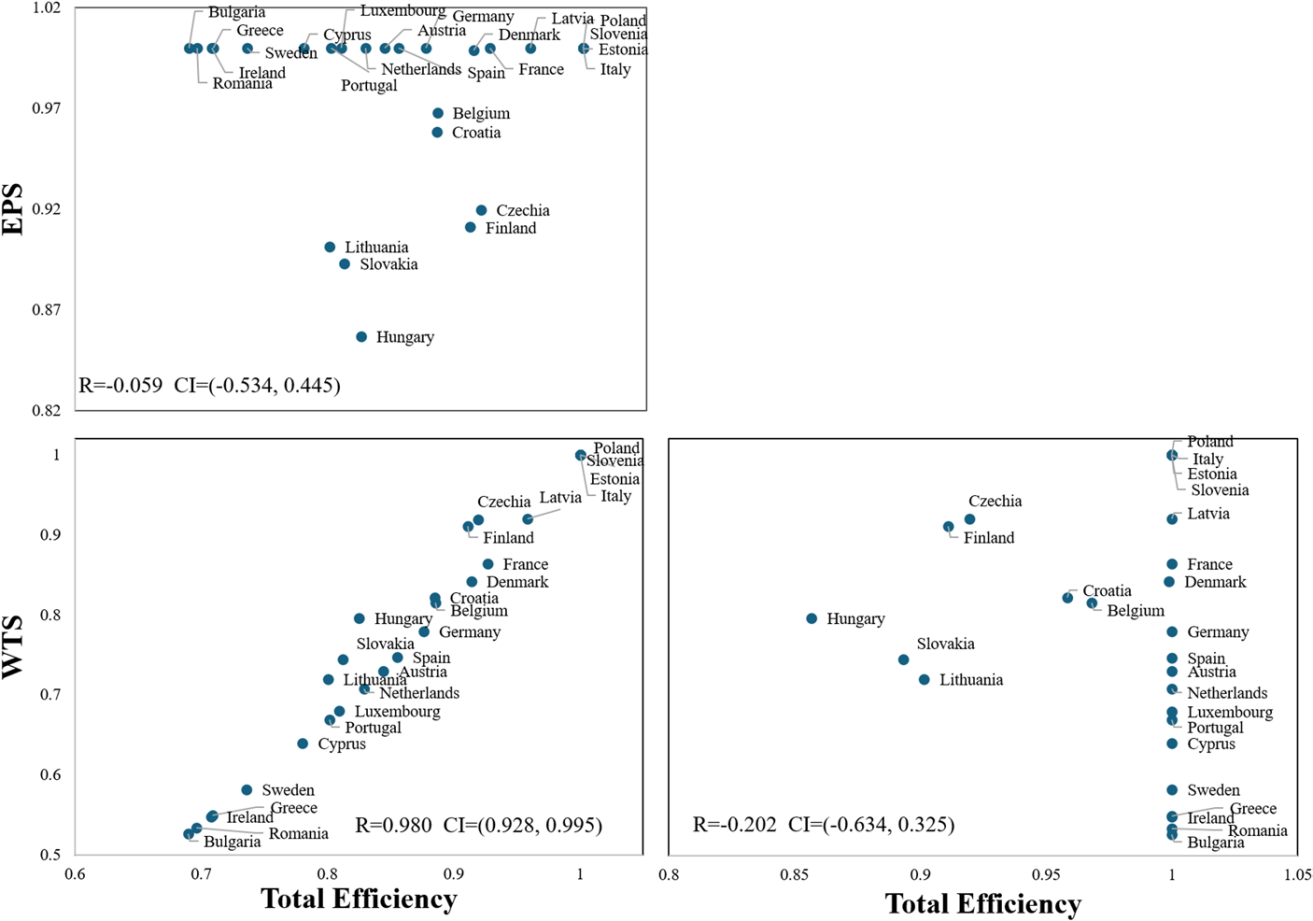
Matrix plot of Total, EPS and WTS efficiency scores

To further explore the relationships between efficiency scores, we analysed the Spearman’s pairwise correlation coefficients among all efficiency scores. Figure 6 provides a matrix plot illustrating these correlations.

First, we find a significant positive correlation of 0.980 (at the 0.01 level)^3^ exists between total efficiency and WTS efficiency. This underscores the substantial influence of waste treatment performance on overall CE efficiency in EU countries. Furthermore, there is not a noticeable correlation between total efficiency and economic EPS efficiency with a correlation coefficient of -0.059 indicating that effective resource utilisation and production efficiency are not important contributors to circularity. In addition , the correlation between EPS efficiency and WTS efficiency is low and statistically insignificant, suggesting minimal interdependence between these two subsystems. In other words, improvements in one do not necessarily drive improvements in the other. Therefore, while WTS is pivotal to overall CE performance, it is essential to address the unique characteristics and challenges of EPS and WTS subsystems. Tailored strategies and interventions are needed for each subsystem to enhance their effectiveness within the broader CE framework.

### Analysis of CE efficiencies

To further analyse the underlying drivers of CE performance in EU countries, we leverage Sovereign ESG data using the ESG score builder tool to calculate Environmental (E), Social (S), and Governance (G) scores for all 26 EU countries. In addition, we apply k-means clustering to classify EU countries into high-, medium- and low-ESG groups. Note that incorporating Sovereign ESG data provides a critical perspective for evaluating how effectively EU countries manage environmental resources, reduce waste, while fostering a sustainable and resilient economy. By integrating sovereign ESG scores into our analysis, we assess the extent to which nations prioritise renewable energy, responsible consumption, and circular resources use; factors that are increasingly valued by all stakeholders. In particularly, linking sovereign ESG scores to CE efficiency offers policymakers valuable insights into whether improvements in environmental, social, and governance aspects are driving enhanced CE performance or if further targeted policies are required. This connection helps to identify gaps and opportunities, enabling a more strategic alignment of sustainability initiatives with CE goals across the EU.

**Fig. 7:**
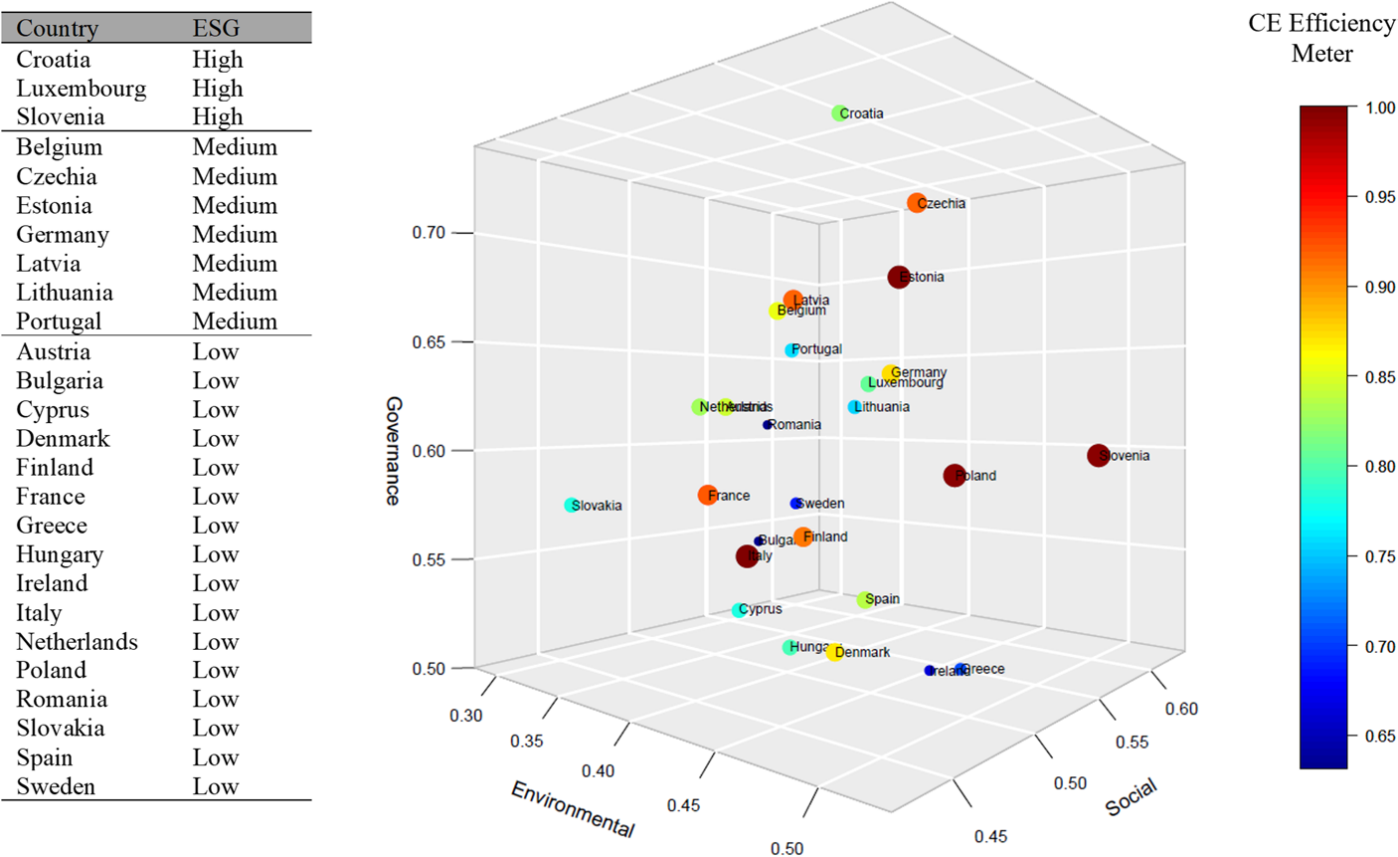
CE Efficiency vs ESG

The 3D scatter plot in Fig. 7 illustrates the relationship between ESG scores and the CE efficiency for each EU country. The size of each oval represents the country's CE efficiency,while its position reflects the ESG scores. The plot reveals a key insight: *a strong ESG standing does not necessarily correspond to high CE efficiency*, *but weak ESG dimensions can impede circularity.* For example, Italy, despite being a large economy with low ESG scores, has achieved perfect CE efficiency, primarily by prioritising the rate of recycled materials in general waste. Similarly, Poland and Estonia rank among the top four EU countries in CE efficiency, even though their ESG rankings are comparatively lower. In contrast, Croatia, with relatively high ESG scores, displays a lower CE efficiency. However, at the lower end of the spectrum, five countries with low ESG scores, Greece, Sweden, Ireland, Bulgaria, and Romania, also rank poorly in CE efficiency. This trend suggests that ineffective Environmental, Social, and Governance dimensions may hinder or decelerate CE initiatives. For these countries, insufficient progress across ESG aspects could act as a barrier to effectively implementing circular practices. In sum, these findings highlight a potential disconnect between ESG performance and CE efficiency, suggesting they may operate independently in some cases. This underscores the need for targeted strategies to address both areas to ensure a more cohesive approach to sustainability and circularity.

Furthermore, comparing the magnitude of economic production and waste treatment activities (Fig. 3) with CE efficiency results from Table 7 and Fig. 5 provides additional insights. The results, sorted by total efficiency scores, with equal weighting assigned to EPS and WTS, reveal clear dynamics between subsystem performances. As Theorem 1, Corollaries 1 and 2 also indicate, countries achieving WTS efficiency also attain total CE efficiency, underscoring the decisive role of waste management in overall performance. By contrast, EPS efficiency alone is insufficient: the Netherlands, for example, is EPS-efficient but WTS-inefficient, rendering it total CE-inefficient. Although the Netherlands has made notable progress in embedding circular principles and decoupling GDP growth from emissions and material use (Foundation and Delivering 2015), further improvements in WTS are required to fully realise its CE potential. These findings emphasise the need for balanced performance across both subsystems to achieve optimal CE outcomes.

In Table 7, it becomes clear that *the scale of activities does not directly translate into higher CE efficiency*. To be more specific, while large-scale activities contribute to circular outputs, countries like Germany, France, and Spain demonstrate that substantial economic activity alone does not ensure optimal CE performance. Similarly, countries such as Netherlands, Luxembourg, Cyprus, Germany, Greece, and Austria, despite achieving high efficiency in economic production metrics, fail to rank among the top for CE efficiency when other environmental factors are considered. This finding highlights a significant challenge: *larger economies can struggle to optimise waste treatment and management despite strong economic production systems*.

As noted in EEA (2023b), more stringent waste management regulations are essential for reconciling economic production with circularity objectives. This disparity between economic scale and circularity efficiency underscores the need for tailored waste management incentives for high-output nations to meet EU circularity objectives (Marques and Teixeira 2022; Robaina et al. 2020; Huang and Hu 2021). Strengthening waste reduction infrastructure and regulations in larger economies will be pivotal for driving the EU’s advancing circularity ambitions while fostering a sustainable balance between economic growth and environmental stewardship.

In our framework, EPS and WTS are complementary yet distinct subsystems within the CE, requiring both to function efficiently to achieve balanced CE performance. For instance, Slovenia emerges as one of the top performers in this study, achieving full efficiency in both EPS and WTS activities by excelling in material use productivity, waste treatment, efficient processes, and the use of recycled materials. Czechia also demonstrates exceptional performance with high material flow efficiency across both EPS and WTS. However, France and Latvia exemplify excellence in circularity, through dominant economic production performance but not through outstanding waste management practices.

Our findings align with prior results, including Sun et al. (2019), which emphasised the integration of economic and waste systems for a comprehensive CE evaluation in China. Likewise, Potkány et al. (2024) highlighted that optimising the efficiency of both production and waste treatment activities is crucial to advancing circularity in Central and Eastern Europe. A multiple-criteria decision-making model introduced in Sassanelli et al. (2019) revealed economic and environmental factors should be treated equally to reach the optimal circularity. Building on these insights, our research demonstrates the interconnected nature of EPS and WTS subsystems. However, an excessive focus on economic output without parallel improvements in waste treatment efficiency can undermine overall CE performance.

### Theoretical implications

This study introduces a comprehensive framework for assessing CE performance at the national level, addressing the challenges posed by data uncertainties that may affect the accuracy of evaluations. By employing an NDEA model with minimal decomposition, where a country's production system is divided into two primary subsystems, EPS and WTS, we identify key drivers of CE and enhance the precision of efficiency evaluations. We also prove that in a circular system, EPS efficiency depends on WTS efficiency, but not vice versa, highlighting the asymmetric yet interdependent relationship between the two subsystems.

In previous studies such as Busu and Busu (2018) and Škrinjarić (2020), CE efficiency was examined but lacked methods to handle unpredictable data inputs. As in our study, incorporating a chance-constrained approach accounts for stochastic variations in economic and environmental data, thereby improving the robustness of CE assessments. DEA analyses catalogued CE indicators but stressed the need for adaptable models to capture CE performance's dynamic nature in Saidani et al. (2019). Our adaptable evaluation methodology helps policymakers refine national circularity policies, especially in balancing economic and environmental goals amid data heterogeneity.

Additionally, the proposed integrated circular chance-constrained NDEA model offers a unique feature for scenario-based analysis tailored to specific policy needs. This approach supports data-informed, context-sensitive decision-making, particularly valuable for policymakers aiming to improve CE metrics in alignment with sustainable development goals. Such a framework not only bolsters country-level CE evaluation but also fosters a data-resilient methodology adaptable to varying levels of data quality and policy objectives.

### Policy implications

The CE is emerging as a critical tool in addressing climate change, and its visibility in internationally recognised indices, such as the Sustainable Development Goals (SDGs) and sovereign ESG frameworks, needs to be strengthened. The integration of CE within these frameworks can incentivise countries to report circularity measures more transparently, thereby supporting more rigorous CE performance analyses.

Furthermore, large EU economies must accelerate circularity initiatives to mitigate their significant environmental impact. Targeted national and international policies, such as those from the EU Commission and taxation incentives, are essential to enforce CE practices in these nations. While rising energy and carbon prices may encourage investments in CE initiatives, dedicated incentives are needed to specifically enhance waste treatment and circularity efforts. Additionally, stricter regulations on waste-generating industries in larger EU economies could reinforce these efforts, helping shift production systems toward more sustainable models that align economic success with environmental responsibility.

Finally, establishing international CE partnerships, including trade agreements, is essential. Such collaborations enable countries to identify and leverage each other's surplus resources, fostering efficient resource sharing that enhances CE outcomes while maximising cost-effectiveness.

## Conclusion

This study makes a theoretical contribution by introducing a novel methodological framework that integrates NDEA with a chance-constrained programming to evaluate CE performance across Europe. Our approach captures the interrelated dynamics of economic production and waste treatment subsystems, accounting for stochastic variables and data uncertainties to provide robust and reliable efficiency estimates. Moreover, we demonstrate that in a circular system, as modelled, perfect EPS efficiency follows from perfect WTS efficiency, but not vice versa, highlighting the asymmetric yet interdependent relationship. These findings highlight that achieving CE efficiency requires a balanced focus on both economic production and waste management. While strong economic output supports circularity, waste treatment efficiency often plays a decisive role in determining overall CE performance. Moreover, the model’s scenario-based flexibility equips policymakers with tools to adapt CE strategies to evolving sustainability goals.

Beyond its theoretical contributions, this study offers several practical insights for stakeholders. First, while a strong ESG sanding does not directly correlate with high CE efficiency, underdeveloped ESG elements can hinder progress. Second, our results reveal that the scale of activities does not inherently translate into higher CE efficiency. Third, larger economies can struggle to optimise waste treatment and management, even with strong economic performance. These findings underscore that robust economic output alone is insufficient to guarantee CE efficiency without optimised waste management practices. Therefore, developing targeted policy interventions is essential for enhancing circularity. These results can support EU policymakers in crafting region-specific CE strategies tailored to the unique strengths and challenges of individual member states.

For large EU economies with substantial environmental footprints, advancing circular activities will require targeted regulations, tax incentives, and stronger enforcement mechanisms. Shifts in consumer behaviour, coupled with rising energy and carbon costs, may drive CE investments, but success requires well-designed coherent incentives and focused interventions. Additionally, international CE alliances and trade agreements also hold potential for improving resource sharing, cost-effectiveness, and circularity.

This study also identifies key avenues for future research. As climate change and resource depletion are global challenges, fostering international collaboration within the CE framework is imperative. Future work could explore the development of models to identify specific industries or international partnerships that can strengthen CE performance. Furthermore, since CE assessments often involve multiple, often conflicting criteria, there is a need for models that can pinpoint inefficiencies and projections at the criterion-level. Such targeted techniques could offer actionable insights and practical solutions to improve CE efficiency across diverse contexts.

## Footnotes

1 https://ec.europa.eu/eurostat/statistics-explained/index.php?title=Glossary:Statistical_classification_of_economic_activities_in_the_European_Community_(NACE).

2 It is important to note that intangible and financial investments are excluded from the scope of INV.

3 Note that confidence interval (CI) is computed at the 99% level.
